# Activated Biochar from Sewage Sludge: A Sustainable Solution for Effective Removal of Emerging Water Contaminants

**DOI:** 10.3390/molecules30173514

**Published:** 2025-08-28

**Authors:** Marina Anastasiou, Vasilios Sakkas, Mohamad Sleiman

**Affiliations:** 1 Institute of Chemistry of Clermont Ferrand, Université Clermont Auvergne, Clermont Auvergne INP, CNRS, ICCF, F-63000 Clermont-Ferrand, France; marina.anastasiou@sigma-clermont.fr; 2 Department of Chemistry, University of Ioannina, 45110 Ioannina, Greece; vsakkas@uoi.gr

**Keywords:** sewage sludge, biochar, adsorption, caffeine, carbamazepine, 17α-ethinyl estradiol

## Abstract

Sewage sludge, a byproduct of wastewater treatment, can be converted into biochar, offering a sustainable solution for waste management and water treatment. Although biochars from biomass have been widely studied, sewage sludge-derived biochars remain underexplored. This study investigated the use of alkaline-treated sewage sludge-derived biochar (AlBC) as an adsorbent for three water pollutants: caffeine (CAF), carbamazepine (CBZ), and 17α-ethinyl estradiol (EE2). A comprehensive analysis was conducted to explore the kinetic and thermodynamic behaviors of these pollutants under varying conditions, such as different adsorbent dosage, temperature, and water matrix values. The AlBCSS showed enhanced surface area and improved adsorption capacity, with EE2 being preferentially adsorbed (q_e_: 9.51 mg g^−1^), followed by CAF (6.12 mg g^−1^) and CBZ (4.58 mg g^−1^). Adsorption followed the Langmuir isotherm for CAF and CBZ, and the Freundlich isotherm for EE2, while kinetics were best described by the pseudo-second-order and Elovich models. Thermodynamic analysis revealed that the adsorption process was spontaneous, primarily driven by physical interactions. Factors such as dosage, temperature, and pollutant concentration influenced adsorption, with no saturation observed at higher concentrations. The natural water matrix had a minimal effect on removal efficiency (40–100%), whereas AlBC exhibited promising results after four adsorption cycles. These results highlight the potential of sewage sludge-derived biochar as a sustainable adsorbent for emerging water pollutants, supporting circular economy practices in wastewater management.

## 1. Introduction

Sewage sludge, the primary solid waste generated by wastewater treatment plants (WWTPs), amounts to millions of tons. In 2022 alone, European Union members produced approximately 4 million tons of sludge (dry weight) [1], with its disposal and processing costs representing nearly 50% of WWTP operational expenses [2]. Consequently, there is growing interest in adopting alternative sludge disposal strategies. Currently, common sludge management methods include incineration, landfilling, and agricultural reuse [3], while, in many cases, only half of the generated sludge is eventually recycled, with the remainder classified as waste [1]. Thermal transformation processes such as gasification, hydrothermal carbonization, and pyrolysis offer promising avenues for converting this waste into biochar, a carbon-rich material with significant potential for environmental applications [4,5].

Biochar, characterized by its high porosity and functional groups, shares similarities with activated carbon but is distinguished by its waste-derived origins, making it an attractive alternative [6,7]. While much of the research on biochar has focused on agricultural and food waste as biomass sources (e.g., wood chips, bagasse, coffee husks) [8,9,10], sewage sludge and manure could be viable sources [11,12]. Compared to biochars from other biomass, sewage sludge-derived biochar tends to have a smaller surface area and lower porosity, which can limit its practical applications [13]. Nonetheless, biochar properties can be optimized through careful selection of feedstock and precise control of pyrolysis conditions [14,15].

Although less extensively studied than other biochar sources, sewage sludge-derived biochar has been explored for various applications, including sorption of nutrients, heavy metals, and organic pollutants, as well as soil amendment and catalysis for pollutant degradation [16,17,18]. The effectiveness of these applications depends largely on the physicochemical properties of biochar, such as its surface area, porosity, chemical stability, and ion exchange capacity [7]. Recent studies have demonstrated that chemical and physical treatments can enhance biochar’s properties for specific environmental applications, particularly in the removal of organic pollutants. Among the most favored treatments, alkaline and acidic activation are used for the chemical modification of biochar, while ball milling is preferred in some studies for achieving a physically modified material [19,20,21,22].

In recent decades, the growing presence of organic micropollutants, including psychotropic substances, pharmaceutical compounds (PhACs), and endocrine-disrupting chemicals (EDCs), in water bodies and wastewater has led to a significant environmental and health challenge considering that, even at low concentrations (<1 µg L^−1^), these pollutants can disrupt biochemical processes and adversely affect aquatic ecosystems [23,24]. For example, caffeine (CAF), a widely consumed compound found in food, drinks, and medicines, is difficult to metabolize and has been shown to cause biochemical alterations in marine species [25,26]. Similarly, studies of endocrine disruptors such as 17α-ethinyl estradiol (EE2) and carbamazepine (CBZ) exhibited that they can also be acutely toxic to aquatic life [27]. Among others, CAF, CBZ, and EE2 are persistent compounds that cannot be removed by conventional wastewater treatments, such as sedimentation and filtration [28], highlighting the need for more efficient treatment technologies.

Among the most promising methods for micropollutant removal, adsorption stands out due to its cost-effectiveness, simplicity, and time efficiency [29]. Materials such as activated carbon, clay, and metal–organic frameworks are commonly used as adsorbents [30,31]. However, activated carbon remains expensive and energy-intensive, underscoring the need for more sustainable and affordable alternatives [32]. While biomass-derived biochars have been extensively studied for their adsorption capabilities, the use of biochar derived from sewage sludge remains underexplored. This study investigated the potential of alkaline-treated sewage sludge-derived biochar (AlBC) as an adsorbent for three organic pollutants—CAF, CBZ, and EE2—with distinct chemical properties. This research systematically examined the kinetic and thermodynamic behaviors of these pollutants under varying conditions, such as different adsorbent dosage and temperature values. The findings provide valuable insights into the potential of sewage sludge-derived biochar as a sustainable, low-cost solution for wastewater treatment, thereby contributing to the principles of a circular economy.

## 2. Results and Discussion

### 2.1. Characterization of the Biochar Samples

Surface functionality, specific surface area, pore volume, and morphology are physicochemical parameters that significantly affect the adsorption capacity of materials. Both untreated (BC) and alkaline-treated biochar (AlBC) were characterized to better understand their properties and confirm the success of the treatment.

**Figure 1:**
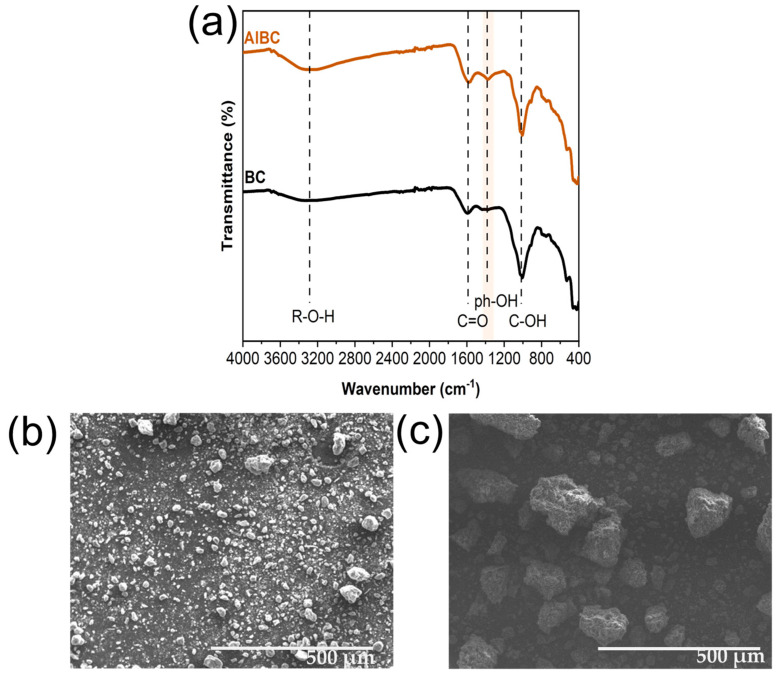
Characterization of biochar samples with ATR-FTIR (**a**) and SEM before (**b**) and after (**c**) alkaline treatment.

The functional groups present on the surfaces of BC and AlBC were explored by ATR-FTIR. As shown in Figure 1a, the surface of the biochar contained various functional moieties. A vibrational band observed at 3327 cm^−1^, although quite weakened due to high-temperature pyrolysis [33], is representative of R-O-H stretching of alcohols. Other oxygenated groups are also present, with bands at 1595 cm^−1^ and 1376 cm^−1^ assigned to C=O stretching of carboxylic acids and phenolic-OH, respectively [34,35]. The enhancement of the bands at 3327 cm^−1^ and 1370 cm^−1^ (shaded area in Figure 1a) is an indication of further functionalization of the initial samples and enrichment with oxygen-containing groups, confirming the successful chemical modification of BC. Moreover, the band observed at 1000 cm^−1^ can be attributed to C-OH stretching vibration. As reported by Yin et al. [34], Si-O vibrations were observed at the same wavenumbers, and their presence was a result of the rich content of minerals and metals preserved from the sewage sludge. Following this observation, bands at 780 cm^−1^ and below 600 cm^−1^ can be considered a result of M-X stretching vibrations (M: metal, X: halogen) [36,37]. Energy-Dispersive Spectroscopy (EDS) conducted on BC confirmed the high carbon (55%) and oxygen content (35%) of the material, but it also revealed the presence of small amounts of Al, Si, P, S, Ca, and Fe, sourced from the selected feedstock, sewage sludge, as shown in Table S1.

The specific surface area (S_BET_) and pore size are critical factors that influence the performance of an adsorbent. Table S2 presents the N_2_ adsorption and desorption isotherms for the BC and AlBC. As shown, the BC exhibited a relatively low S_BET_ of 28.9 m^2^ g^−1^, consistent with previously reported values [38,39]. For instance, Kalderis et al. [40] reported an S_BET_ of 25.6 m^2^ g^−1^ for sewage sludge biochar in their study on the adsorption of 2,4-dichlorophenol. These findings suggest that biochar derived from sewage sludge generally has a low specific surface area, which may limit its adsorptive capacity. To address this limitation, alkaline treatment has been shown to significantly improve S_BET_ and enhance the material’s porosity [41]. In this study, NaOH treatment resulted in a threefold increase in S_BET_. The NaOH-treated biochar, AlBC, also exhibited enhanced N_2_ adsorption and increased pore volume, as illustrated in Figure S1, supporting the hypothesis that the treatment created a more porous structure.

As shown in Figure 1b,c, both BC and AlBC exhibited considerable morphological heterogeneity, consisting of irregular, non-spherical particles. Notably, AlBC particles had a rougher surface, which may enhance its adsorptive capacity, and a larger particle size compared to BC. This observation is further supported by a particle size analysis, which revealed DX50 values of 8.19 µm and 24.1 µm for BC and AlBC, respectively. The increase in size was likely due to the agglomeration of smaller particles during the activation process. Although alkaline treatment of biochar has been widely used as a modification method [42,43,44,45], there are very few studies on its effect on particle size. Liu et al. [46] and Yang et al. [47] utilized alkaline agents to improve their material’s adsorptive capacities for the removal of methylene blue and ammonium, respectively. As reported, an increase in the particle size of biochar was noted after treatment at the scale of a few nanometers in the first case and in a range of micrometers in the second case, as is evident from the size distribution results. These reports support the findings of this study regarding the increased particle size of biochar after treatment.

Another set of factors with critical influence in the performance of biochar is pH and zeta potential. Suspensions of BC and AlBC in deionized (DI) water exhibited pH values of 7.10 and 7.69, respectively. The zeta potential increased from −26.18 ± 1.08 mV for BC to −32.69 ± 0.9 mV for AlBC, as shown in Table S2. These values confirm the negatively charged surfaces of the biochars and their high stability in suspension, while also suggesting an increased concentration of oxygenated functional groups in the treated biochar.

### 2.2. Adsorption Kinetics Study

**Figure 2:**
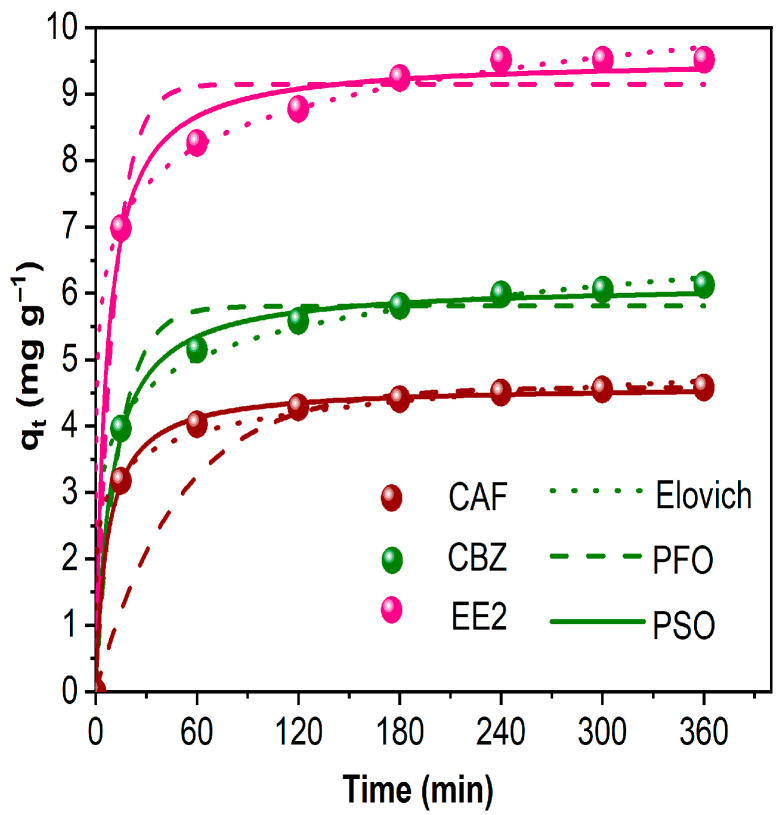
Adsorption kinetics of CAF, CBZ, and EE2 on AlBC with Pseudo-First-Order (dashed line), Pseudo-Second-Order (continuous line), and Elovich fitting (dotted line) (C_AlBC_: 1 g L^−1^; C_contaminant_: 10 mg L^−1^; 400 rpm; 25 °C).

The kinetic profiles of CAF, CBZ, and EE2 adsorption onto AlBC were evaluated using three models: the Pseudo-First Order—PFO, Pseudo-Second Order—PSO, and Elovich, as depicted in Figure 2. Under the experimental conditions, all three contaminants exhibited rapid adsorption within the first 60 min, likely due to initial physical adsorption onto the biochar’s surface. After 60 min, the adsorption rate decreased, and equilibrium was reached at 360 min. This slower second phase of adsorption may be attributed to the filling of the inner pores of the biochar, as most of the available surface active sites were likely initially occupied by the contaminants.

The adsorption of CAF, CBZ, and EE2 on AlBC was efficient, with EE2 showing the highest removal efficiency. The equilibrium adsorption capacities (q_e,exp_) for the three contaminants were 4.58, 6.12, and 9.51 mg g^−1^, respectively. The higher removal of EE2 can be attributed to its higher lipophilicity (LogK_ow_ = 3.67), which likely enhances its affinity for biochar, a highly carbonaceous material. Similar adsorption capacities have been reported in the literature for biochars employed for the adsorptive removal of these three contaminants. More specifically, Anastopoulos et al. (2020) [48] reported an adsorption capacity of 5.35 mg g^−1^ (at 25 °C) for the removal of CAF using oxidized pine needle-derived biochar, a value that is in the same range as the results reported in this study. However, several studies have reported q_e_ values in the range of hundredths for food waste-derived biochars that possess equally high surface area values [49,50,51]. In the case of CBZ, Zhang et al. [52] reported a maximum adsorption capacity of 118.4 mg g^−1^ when using wastewater algae-derived biochar, while, for EE2, Shin et al. [53] reported q_e_ values equal to 18.73 mg g^−1^ for alkali-modified biochar from spent coffee grounds, a value that is almost double those reported in this study.

Among the three kinetic models applied, the PSO and Elovich models provided the best fit to the experimental data, as evidenced by the high R^2^ values (R^2^ > 0.99), the statistical parameters (Adjusted R^2^, Residual Sum of Squares, Reduced Chi-Square) exhibited in Table S3, and the close agreement between the experimental and calculated q_e_, as shown in Table 1. Both the PSO and Elovich models are suitable for describing adsorption processes that involve chemisorption during the rate-determining step [54]. These findings are consistent with previous studies, which have also reported that the PSO model effectively describes the adsorption of CAF, CBZ, and EE2 on biomass-derived biochar [55,56].

### 2.3. Adsorption Isotherm Study

**Figure 3:**
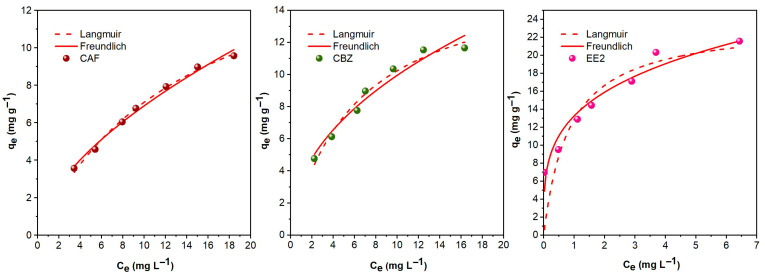
Langmuir and Freundlich adsorption isotherms of CAF, CBZ, and EE2 on AlBCSS (C_AlBC_: 1 g L^−1^; C_contaminant:_ 7–28 mg L^−1^; 400 rpm; 25 °C).

The Langmuir and Freundlich models were applied to better understand the adsorption behavior and equilibrium characteristics of the contaminants, as shown in Figure 3. The Langmuir model assumes monolayer adsorption on a homogeneous surface, whereas the Freundlich model describes multilayer adsorption on a heterogeneous surface with active sites of varying intensities.

In this study, the Langmuir model best described the adsorption behavior of CAF and CBZ on AlBC, according to the adsorption isotherm studies (Table S4). The monolayer adsorption capacities (q_e_) were 17.05 mg g^−1^ for CAF and 16.56 mg g^−1^ for CBZ. The obtained values were higher than the adsorption capacities of white pine needle biochar for the adsorption of CAF, as reported by Oginni and Singh [57], and comparable to the adsorption capacity of biochar for the adsorption of CBZ, as reported by Całus-Makowska et al. [58]. These findings prove that the efficiency of AlBC is comparable, if not superior, to other biochars already reported in the literature [59,60].

Apart from the maximum adsorption capacity, the Langmuir model provides information regarding the affinity between the adsorbent and adsorbate through the Langmuir isotherm constant K_L_. High K_L_ values indicate a stronger interaction between the adsorbent and adsorbate. As presented in Table 2, CBZ exhibited a higher K_L_ (0.161) than CAF (0.071). This result is consistent with the fact that CBZ is a more lipophilic molecule (LogK_ow_: 2.45) than the hydrophilic CAF and thus shows a greater affinity for the hydrophobic AlBC [61].

The adsorption of EE2, on the other hand, adhered to the Freundlich model, as indicated by the higher R^2^ values summarized in Table 2. EE2 has the most distinct properties among the studied contaminants, with a higher molecular weight and lipophilicity (high LogK_ow_). As observed, its adsorption was higher than that of CAF and CBZ, with nearly double the q_e_ values. These results are in agreement with those reported in the literature, where the Freundlich model best describes the experimental results compared to the Langmuir [62].

The key parameters derived from the Freundlich model include the heterogeneity factor ($n_{F}$) and 1/$n_{F}$. An $n_{F}$ value between 1 and 10 suggests that the adsorption is favorable, with higher values of n indicating a strong interaction between the adsorbent and adsorbate. For EE2, the $n_{F}$ value was 3.82, indicating favorable adsorption. This value is higher than those reported by Ahmed et al. [63] for the removal of endocrine disruptors, including EE2, by Eucalyptus globulus wood biochar ($n_{F}$ between 0.17 and 0.27) and by Vieira et al. [64], where the $n_{F}$ value was 1.107 for the adsorption of EE2 from fungiculture waste-derived biochar.

### 2.4. Thermodynamic Adsorption Parameters

While kinetic and isotherm parameters have been extensively studied in adsorption research, thermodynamics remain less explored. Thermodynamic analysis of the adsorption process provides insight into the nature of adsorption (whether physical or chemical) and validates the predictions made by isotherm models based on the values of enthalpy change (ΔH^0^), Gibbs free energy (ΔG^0^), and entropy change (ΔS^0^). To calculate these parameters, the dimensionless thermodynamic equilibrium constant (K^0^) is used in the Van ‘t Hoff equation, ensuring correct units for ΔG^0^ [65], in contrast to studies that directly use the Langmuir or Freundlich constants. Table 3 presents the calculated thermodynamic parameters for the adsorption of CAF, CBZ, and EE2 onto AlBC. The ΔH^0^ values increased from CAF to EE2. ΔH^0^ values below 20 kJ mol^−1^, such as those calculated for CAF (ΔH^0^ = −15.99 KJ mol^−1^), CBZ (ΔH^0^ = 2.30 KJ mol^−1^), and EE2 (ΔH^0^ = 3.09 KJ mol^−1^), indicate physisorption. The negative ΔH^0^ value for CAF indicates an exothermic process, whereas the positive ΔH values for CBZ and EE2 suggest endothermic processes. For all three contaminants, the entropy change (ΔS^0^) was positive, with values increasing from CAF (60.32 J mol^−1^ K^−1^) to EE2 (176.25 J mol^−1^ K^−1^), indicating greater disorder at the solid–liquid interface. The negative ΔG values for all three contaminants confirmed the spontaneity of the adsorption process. Notably, as the temperature increased from 10 to 40 °C, the magnitude of the negative ΔG^0^ values became more pronounced, further supporting the endothermic nature of the adsorption processes, particularly for CBZ and EE2. It is important to note that the obtained results are in agreement with previously reported thermodynamic values for the adsorption of CAF [66] and CBZ [67] on lignocellulosic biomass-derived biochar. As expected, owing to the variation in the chemical composition of the adsorbent, the compared values are not arithmetically identical; however, there is agreement on their range and the conclusions drawn based on them. In the case of EE2, information on thermodynamic values was not found regarding the adsorption of the compound on carbon-rich materials with properties similar to those of biochar.

### 2.5. Effect of Various Parameters

#### 2.5.1. Effect of Dosage

**Figure 4:**
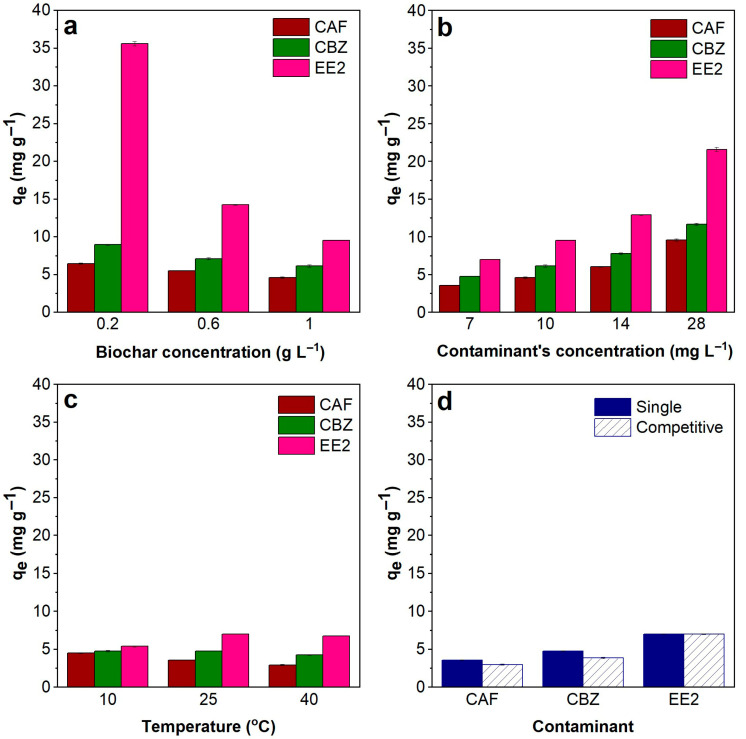
The effect of (**a**) biochar dosage, (**b**) contaminant dosage, (**c**) temperature, (**d**) competitiveness on the adsorption of CAF, CBZ, and EE2 on AlBC.

Several experimental factors influence the efficiency of biochar in adsorption processes, with biochar concentration being one of the most significant factors. In this study, three concentrations of biochar (0.2, 0.6, and 1 g L^−1^) were tested for their ability to remove a fixed concentration of contaminants (10 mg L^−1^) from a solution. As shown in Figure 4a, an increase in biochar concentration led to a decrease in adsorption capacity. Specifically, this decrease was highly evident in the case of EE2, where q_e_ dropped from 35.58 mg g^−1^ to 9.51 mg g^−1^. For CAF and CBZ, the phenomenon was milder because the decrease in the adsorption capacity was below 35% of the maximum q_e_. Although a higher biochar concentration resulted in a reduced q_e_, it led to higher removal efficiencies. The %R for EE2 increased from 71% to 95%, whereas the %R for CAF and CBZ exhibited more pronounced improvements, rising from 13% to 46% for CAF and from 18% to 61% for CBZ. This suggests that optimizing biochar loading can lead to the near-total removal of contaminants such as EE2. At higher biochar concentrations, the availability of active sites is greater, resulting in an increase in %R. However, particle agglomeration may occur, limiting the accessibility of contaminant molecules to the internal pores of the biochar, leading to reduced q_e_ values [68]. Similar results were observed by other researchers, where an increased concentration of sludge-derived biochar led to higher removal efficiencies for Methylene Blue but lowered the adsorption capacities [69]. Similar effects were reported by Zeghioud et al. [70] in their study on the removal of methylene blue and CBZ from aqueous solutions using biochars derived from beech and flax.

Conversely, increasing the contaminant concentration from 7 to 28 mg L^−1^ resulted in an increased adsorption capacity, which was observed for all of the contaminants, as depicted in Figure 4b. Even though a slight decrease was observed in the %R, removal efficiencies were similar, with the lowest values being 35–40% for CAF and CBZ and the highest being 75% for EE2. The same effect was reported by other studies [71], where higher concentrations in the solution led to higher q_e_ values until saturation of the adsorbent was observed, as a result of the occupation of the available active sites. In our case, given that these concentrations were much higher than typical environmental concentrations, it can be concluded that the adsorbent did not reach saturation even under extreme conditions.

#### 2.5.2. Effect of Temperature

Temperature plays a critical role in adsorption efficiency. To assess its effect, the removal percentages (%R) of the three contaminants were evaluated at an initial concentration of 7 mg L^−1^ under three different temperatures (10, 25, and 40 °C), as presented in Figure 4c.

For caffeine (CAF), adsorption decreased with increasing temperature, from 4.47 mg g^−1^ at 10 °C to 2.92 mg g^−1^ at 40 °C. This trend aligns with the weak and exothermic nature of the adsorption process observed in thermodynamic studies. These findings are consistent with previous research indicating that adsorption is exothermic, with higher temperatures leading to reduced adsorption capacities [48,72]. Similarly, Anastopoulos and Pashalidis [73] reported a decline in adsorption capacity for caffeine on oxidized carbon derived from Luffa cylindrica, where the maximum adsorption capacity (*q*_max_) decreased from 59.9 to 49.3 mg g^−1^ as temperature increased from 298 K to 323 K.

In contrast, carbamazepine (CBZ) removal was largely unaffected by temperature, with only a slight decrease in adsorption from 4.47 mg g^−1^ at 10 °C to 4.22 mg g^−1^ at 40 °C. This observation is in agreement with findings by Zhang et al. [52], who noted a decrease in CBZ adsorption by algae-derived biochar with increasing temperature. Among the three pollutants, the adsorption capacity for 17α-ethinylestradiol (EE2) increased from 5.36 mg g^−1^ at 10 °C to 6.98 mg g^−1^ at 25 °C, followed by a slight decline to 6.72 mg g^−1^ at 40 °C. This behavior supports the endothermic nature of EE2 adsorption (ΔH^0^ > 0). Similar results were reported by Prokić et al. [74], who observed increased adsorption of EE2 using activated carbonized hydrothermal carbon as the system temperature rose from 25 °C to 55 °C.

#### 2.5.3. Effect of Co-Presence of the Contaminants

All the above experiments were performed under single-contaminant conditions. However, in real-world scenarios, multiple contaminants often coexist. To mimic this complexity, competitive adsorption experiments were conducted and compared with single-contaminant systems to evaluate the influence of coexisting contaminants on adsorption performance. As illustrated in Figure 4d, the adsorption capacities of CAF and CBZ were slightly reduced under competitive conditions, while EE2 removal remained largely unaffected. This suggests that EE2 exhibits a higher affinity for AlBC compared to CAF and CBZ. The observed reduction in CAF and CBZ adsorption may be attributed to the preferential occupation of active sites by EE2 molecules. Once adsorbed, EE2 may hinder further access to adsorption sites for CAF and CBZ, thereby decreasing their equilibrium adsorption capacities (q_e_). Despite the presence of multiple contaminants, AlBC demonstrated effective adsorption performance, achieving removal efficiencies ranging from 40% to 100% across all pollutants under competitive conditions.

### 2.6. Application in Real Samples

To evaluate the performance of AlBC under more realistic conditions, its adsorption efficiency for CAF, CBZ, and EE2 was tested using a natural water sample collected from the Artière River, located in the Puy-de-Dôme region of France. The chemical characteristics of the sample are detailed in Table S5. Total Organic Carbon (TOC) measurements revealed that most of the carbon content was inorganic in nature (23.83 out of 30.95 mg L^−1^). Additionally, fluorescence and UV–Vis spectra (Figure S2) confirmed the presence of only a small fraction of organic matter.

**Figure 5:**
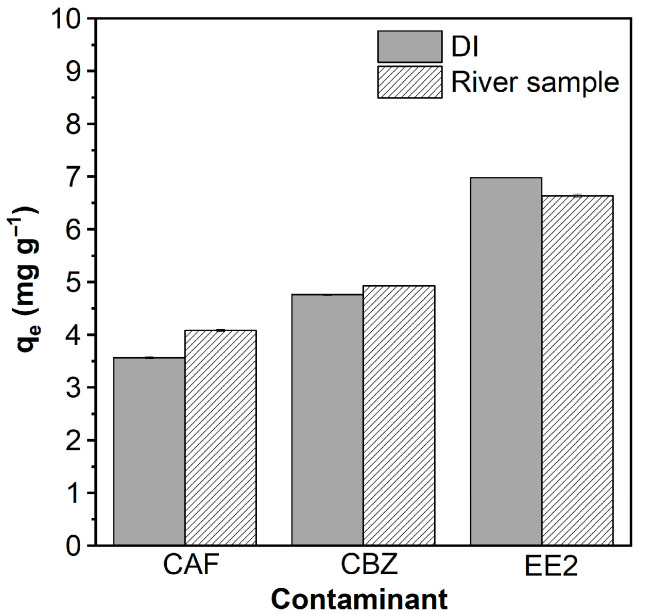
Effect of matrix on the adsorption capacity (q) of AlBC for CAF, CBZ, and EE2 in DI and natural water samples.

Single-contaminant adsorption tests in river water showed comparable or even improved removal efficiencies relative to those in deionized (DI) water, as illustrated in Figure 5. Specifically, the adsorption capacity for CAF increased from 3.56 to 4.10 mg g^−1^, while CBZ showed a slight increase from 4.75 to 4.93 mg g^−1^. EE2 exhibited a minor decrease, from 6.98 to 6.64 mg g^−1^. These promising results suggest that AlBC maintains effective performance under natural water conditions, with adsorption efficiencies similar to or slightly higher than those observed under controlled laboratory settings.

Although Natural Organic Matter can often inhibit adsorption, the low organic content in this river water sample did not significantly affect AlBC’s performance. This finding supports the potential application of AlBC in real-world environments, such as rivers and lakes, and highlights the importance of future studies exploring its use in more complex matrices, including wastewater effluents.

### 2.7. Reusability

**Figure 6:**
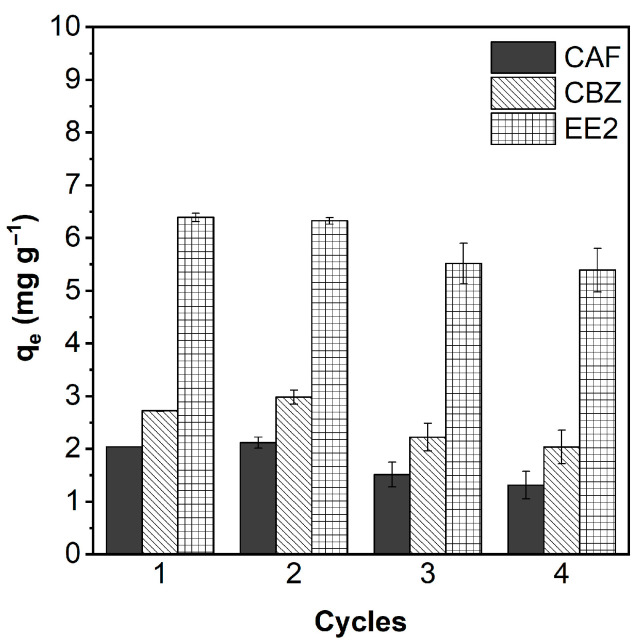
Reusability study of AlBC and its adsorptive capacity for CAF, CBZ, and EE2 after four cycles.

The reusability of AlBC is a critical parameter for evaluating its long-term stability and feasibility in wastewater treatment applications. To assess this aspect, adsorption experiments were carried out under optimal conditions with the simultaneous presence of CAF, CBZ, and EE2. After each adsorption cycle, AlBC was recovered by filtration and thoroughly washed with ethanol and deionized water to remove loosely bound contaminants [75], then reused in a subsequent cycle. This process was repeated for four consecutive cycles, and the results are shown in Figure 6.

AlBC exhibited high reusability. After four cycles, the most notable decrease in adsorption capacity was observed for CAF, with a 36% reduction in q_e_. EE2 displayed the smallest reduction, with only a 15% decline compared to the initial cycle. A more pronounced decrease in performance was observed after the second cycle, but this did not significantly affect the overall efficiency.

These results demonstrate that AlBC can effectively retain its adsorption capabilities over multiple cycles. Comparable studies have reported similar trends: Behera et al. noted a 40% retention of Rhodamine B adsorption on seed-derived char after four cycles [76], while Zayatt et al. observed a 70% retention of CBZ adsorption on biochar from waste date pits after four cycles [67]. Relative to these, AlBC exhibits competitive reusability, underscoring its potential as a sustainable and effective adsorbent for water purification.

### 2.8. Proposed Mechanism of Adsorption

Understanding the adsorption mechanism is essential for optimizing the performance of an adsorbent. The nature of both the adsorbent and the adsorbates determines the interactions—whether physical or chemical—that govern adsorption. In this study, the AlBC, derived from sewage sludge and chemically activated with NaOH, was highly carbonaceous and contains aromatic rings and surface oxygen-containing functional groups such as –OH and –COOH. BET analysis revealed a moderate surface area (86.41 m^2^ g^−1^) with predominantly mesoporous characteristics. The surface was also found to be negatively charged under the experimental conditions.

The target contaminants—CAF, CBZ, and EE2—are all aromatic organic molecules. Therefore, π–π interactions between the aromatic rings of the pollutants and the AlBC surface were highly likely [77]. Additionally, lipophilicity plays a significant role in the adsorption process, increasing from CAF to CBZ and then to EE2. The greater affinity of EE2 for AlBC can be attributed to its higher lipophilicity and the development of hydrophobic interactions. CBZ, being moderately lipophilic, is also likely to be adsorbed via similar interactions [78]. In contrast, the more hydrophilic CAF showed weaker interactions, as reflected by its comparatively lower adsorption capacity.

However, CAF has the smallest molecular weight among the three contaminants, which may facilitate its diffusion into the inner pores of AlBC through pore-filling mechanisms. Due to their pKa values, all three contaminants are present in neutral form under the optimal working conditions, minimizing the possibility of electrostatic interactions with the negatively charged AlBC surface.

ATR-FTIR analysis of AlBC after adsorption (Figure S3) did not show any significant shifts in characteristic bands, indicating that direct chemical bonding with surface functional groups is unlikely. This, together with thermodynamic data, supports the hypothesis that the adsorption process is primarily governed by physical interactions—mainly π–π stacking, hydrophobic interactions, and pore filling. Although elucidating the precise mechanism was not the primary focus of this study, the proposed pathways are supported by both experimental results and the previously published literature on micropollutant adsorption using biochar-based materials [79].

### 2.9. Comparison with Other Carbon-Based Adsorbents

To benchmark the performance of AlBC, the results of this study were compared with previously reported carbon-based adsorbents, primarily biochars, used for the removal of CAF, CBZ, and EE2, as presented in Table 4. Most of these adsorbents underwent physical or chemical treatments to enhance their adsorption efficiency, such as ball milling or activation with acids and alkalis. In this work, NaOH was selected as the chemical activating agent due to its well-known efficiency, lower corrosiveness, and cost-effectiveness compared to commonly used alternatives like KOH.

In addition to the advantages of the activating agent, the choice of feedstock for AlBC offers notable benefits. While many studies utilize lignocellulosic materials such as wood chips or coffee grounds—which may have competing applications like composting or energy recovery—sewage sludge, the source material for AlBC, is a genuine waste product. Converting it into biochar not only reduces the volume of waste requiring disposal but also produces a valuable material for environmental remediation.

Despite its relatively small surface area and moderate adsorption capacity—particularly for CBZ—AlBC demonstrated consistent performance under varying environmental conditions, including temperature fluctuations and complex water matrices. This reliability, combined with its sustainability and cost-effectiveness, makes AlBC a promising and competitive alternative for real-world water treatment applications.

## 3. Materials and Methods

### 3.1. Reagents

Biochar derived from sewage sludge and pyrolyzed at 550 °C (referred to as BC) was obtained from the United Kingdom Biochar Research Centre (UKBRC) in Edinburgh, United Kingdom. Caffeine (CAF, purity 99%), carbamazepine (CBZ, purity > 98%), and 17α-ethinylestradiol (EE2, purity ≥ 98%) were purchased from Sigma-Aldrich (Darmstadt, Germany). High-performance liquid chromatography (HPLC)-grade organic solvents—methanol (CH_3_OH) and acetonitrile (C_2_H_3_N)—along with hydrochloric acid (HCl, 37%) were also procured from Sigma-Aldrich. Formic acid (CH_2_O_2_, 98%) and sodium hydroxide (NaOH) were obtained from VWR (Rosny-sous-Bois, France). The physicochemical properties of the selected contaminants are summarized in Table 5.

### 3.2. Biochar Preparation, Treatment, and Characterization

The obtained biochar (BC) was milled for 1 min using an analytical mill (IKA A10, IKA-Werke GmbH & Co. KG, Staufen, Germany) to obtain a fine powder. A 1 g amount of the milled biochar was then treated with 2 M NaOH at a ratio of 1 g to 50 mL for 24 h. The treated biochar (referred to as AlBC) was recovered by filtration, thoroughly washed with deionized (DI) water until it reached neutral pH, and dried overnight at 100 °C. After drying, the biochar was ground into a fine powder using an agate mortar and pestle, and stored in a sealed container for subsequent use.

### 3.3. Biochar Characterization

The biochar samples were characterized for particle size distribution using granulometry (Mastersizer 3000, Malvern Panalytical, Palaiseau, France). The specific surface area and porosity were determined using Brunauer–Emmett–Teller (BET) N_2_ adsorption–desorption isotherms, measured with an automated surface area analyzer (ASAP 2020, Micromeritics, Norcross, GA, USA). The morphology and elemental composition of the biochar were analyzed using scanning electron microscopy (SEM) coupled with energy-dispersive spectroscopy (EDS) (Tabletop SEM SH-4000M, Hirox Europe, Limonest, France). Surface functional groups were identified via attenuated total reflectance Fourier-transform infrared spectroscopy (ATR-FTIR) using a Spectrum Two spectrometer (PerkinElmer, Waltham, MA, USA) in the wavenumber range of 400–4000 cm^−1^. The pH of biochar suspensions in deionized water was measured using a HANNA edge^®^ pH meter (HANNA Instruments, Lingo Tanneries Cedex, France), while zeta potential measurements were performed using Laser Doppler Electrophoresis (LDE) with a dynamic light scattering and zeta potential analyzer (AMERIGO, Cordouan Technologies, Pessac, France).

### 3.4. Characterization of Natural Water Sample

A natural water sample was collected from the Artière River in the Puy-de-Dôme region of France. The pH of the sample was measured immediately after collection using a HANNA edge^®^ pH meter (HANNA Instruments, Lingo Tanneries Cedex, France). The sample was then filtered using a 5 μm PVDF membrane, and turbidity was measured both before and after filtration with a turbidimeter (TB350 WL, Lovibond, Dortmund, Germany). Total Organic Carbon (TOC) and Inorganic Carbon (IC) concentrations were quantified using a TOC analyzer (TOC-L, Shimadzu, Kyoto, Japan). Natural Organic Matter (NOM) in the sample was assessed using UV–Vis spectroscopy (Cary 300, Agilent Technologies, Santa Clara, CA, USA) and fluorescence spectroscopy (Cary Eclipse, Varian, Palo Alto, CA, USA).

### 3.5. Batch Adsorption Experiments

Stock solutions of contaminants (1000 mg L^−1^) were prepared by dissolving 0.5 g of each contaminant in 50 mL volumetric flasks. DI water was used as a solvent for caffeine, and methanol for carbamazepine and EE2. To prepare working solutions with the desired contaminant concentrations, appropriate volumes of the stock solution were diluted with DI water.

Batch adsorption experiments were conducted in duplicate using 250 mL double-walled Pyrex beakers, designed to allow water circulation for temperature control.

Adsorption capacity (q_t_) and removal efficiency (%R) were calculated by the following equations:

(1) $$q_{t}\;=\;\frac{C_{0}-C_{t}}{m}V$$

(2) $$\%R=\frac{C_{0}-C_{t}}{C_{0}}100$$

where q_t_ is the amount of adsorbed substance at time t expressed in mg of adsorbed substance/g of adsorbent, C_0_ is the liquid-phase initial concentration of the adsorbate (mg L^−1^), C_t_ is the liquid-phase concentration of the adsorbate at equilibrium (mg L^−1^), V is the volume of adsorbate solution (L), and m is the mass of the adsorbent (g).

### 3.6. Adsorption Kinetics Study

The kinetics of adsorption were monitored over a period of 360 min at a contaminant concentration of 10 mg L^−1^. Working conditions included a biochar dosage of 1 g L^−1^, total volume of 50 mL, agitation at 400 rpm, a contact time of 360 min, neutral pH, and a temperature of 25 °C.

To better understand the adsorption process, three kinetic models were evaluated: the Pseudo-First-Order (PFO) model [88], the Pseudo-Second-Order (PSO) model [89], and the Elovich model [88].

The PFO model:

q_t_ = q_e_ (1 − e^−k1 t^) (3)

The PSO model:

(4) $$q_{t}=\frac{k_{2}{q_{e}}^{2}t}{1+k_{2}q_{e}t}$$

For the Elovich model, q_t_ is calculated by the following equation:

(5) $$q_{t}=\frac{1}{\beta }\times \ln\;\left(\alpha \beta \right)+\frac{1}{\beta }ln\;(t)$$

where t is the contact time (min), q_e_ the mg of adsorbed substance per g of the adsorbent at equilibrium (q_e_), k_1_ the adsorption rate constant for Pseudo-First-Order reaction kinetics (min^−1^), k_2_ the adsorption rate constant for Pseudo-Second-Order reaction kinetics (g mg^−1^ min^−1^), α the initial adsorption (mg g^−1^ min^−1^), and β the desorption rate constant (g mg^−1^).

### 3.7. Adsorption Isotherm Study

For the adsorption isotherm study, seven contaminant concentrations (7, 10, 14, 16, 20, 24, and 28 mg L^−1^) were tested under the same experimental conditions as for the kinetics.

The Langmuir [90] and Freundlich [91] models were applied to determine which better fits the experimental data and determines the maximum adsorption capacity best.

The Langmuir isotherm:

(6) $$q_{e}=q_{\max}\frac{K_{L}C_{e}}{1+K_{L}C_{e}}$$

The Freundlich isotherm:

q_e_ = K_F_C_e_^1/n^ (7)

where C_e_ is the liquid-phase concentration of the adsorbate at equilibrium (mg L^−1^ or mol L^−1^), q_max_ is the maximum adsorption capacity of the adsorbent (mg g^−1^), K_L_ is the Langmuir constant indicative of the strength of the contaminant’s adsorption on the surface of the adsorbent, K_F_ is the adsorption affinity-related parameter [(mg kg^−1^) (mg L^−1^)^1/n^], and n is the nonlinear coefficient.

### 3.8. Thermodynamic Study

To investigate the thermodynamics of the adsorption process, experiments were performed at three different temperatures (10, 25, and 40 °C) while keeping all other conditions constant as for the adsorption isotherms.

The thermodynamic parameters, Gibbs free energy change (ΔG^0^), and enthalpy and entropy changes (ΔH^0^ and ΔS^0^) were determined by the following equations, which were applied according to [92]:

(8) $$\Delta G^{0}=-RTln(K^{0})$$

(9) $$ln(K^{0})=\frac{{\Delta S}^{0}}{R}-\;\frac{{\Delta H}^{0}}{RT}$$

(10) $$K^{0}={K_{L}M}_{adsorbate}10^{3}55.5$$

where R is the universal gas constant with value 8.314 (J mol^−1^ K^−1^), T is the temperature in Kelvin (K), and $K^{0}$ is the standard equilibrium constant (dimensionless) that results from the multiplication of $K_{L}$ with M_adsorbate_, the molar mass of the adsorbate (g mol^−1^), 10^3^ Kg and 55.5.

### 3.9. Effect of Different Parameters

The effect of biochar dosage on adsorption was evaluated at three concentrations (0.2, 0.6, and 1 g L^−1^) while keeping the following parameters constant: a contaminant concentration of 10 mg L^−1^, total volume of 50 mL, agitation at 400 rpm, a contact time of 360 min, neutral pH, and a temperature of 25 °C. The influence of temperature on adsorption was examined at 10 °C, 25 °C, and 40 °C using a biochar dosage of 1 g L^−1^ and a contaminant concentration of 7 mg L^−1^ under the same conditions as those for the adsorption isotherms. Competitive adsorption of the three contaminants was studied using the same parameters as those applied in the single-contaminant adsorption experiments.

### 3.10. Matrix Effect

To assess the efficiency of biochar in complex matrices, a natural river water sample was collected. Unlike the deionized (DI) water used in previous experiments, river water contains various inorganic and organic species, including Natural Organic Matter (NOM). For this experiment, the lowest contaminant concentration (7 mg L^−1^) was selected under the same conditions as in the kinetics study. Samples were collected at 0, 15, and 60 min, followed by additional sampling at 60 min intervals up to 360 min.

### 3.11. Determination and Quantification of the Contaminants

Samples collected during the adsorption experiments were filtered through 0.45 µm PTFE filters. Contaminant concentrations in the filtrate were determined by high-performance liquid chromatography with a diode array detector (HPLC-DAD). Specifically, analyses were performed using an Agilent 1290 series HPLC system equipped with a reverse-phase NUCLEODUR C18 ec column (150 × 4.6 mm) and a mobile phase consisting of acetonitrile (ACN) and 0.3% formic acid in water (50:50 *v*/*v*). The injection volume was 10 µL, the column temperature was maintained at 40 °C, and the flow rate was set at 1 mL min^−1^. Detection wavelengths were 274 nm for caffeine, 286 nm for carbamazepine, and 280 nm for EE2. The limits of detection (LOD) and quantification (LOQ) are provided in Table S6.

## 4. Conclusions

In this study, sewage sludge-derived biochar (BC) was successfully activated using NaOH, resulting in the incorporation of additional oxygen-containing functional groups and a threefold increase in specific surface area (AlBC). Adsorption behavior varied by contaminant: CAF and CBZ followed the Langmuir isotherm model, indicating monolayer adsorption, while EE2 was better described by the Freundlich model, suggesting heterogeneous surface interactions. Kinetic modeling showed that the Pseudo-Second-Order and Elovich models best fitted all three contaminants, and thermodynamic analysis confirmed that adsorption was spontaneous and primarily driven by physical interactions.

AlBC’s adsorption performance was influenced by biochar dosage and temperature but not significantly limited by higher contaminant concentrations. In multicomponent systems, EE2 was preferentially adsorbed, consistent with its higher lipophilicity (LogK_ow_), while CAF and CBZ were only slightly affected. Notably, AlBC maintained high adsorption efficiency in natural river water, comparable to its performance in deionized water, indicating its robustness under environmentally relevant conditions.

Reusability tests demonstrated that AlBC retained considerable adsorption capacity over multiple cycles, with EE2 showing the least decline. Although its overall adsorption capacity is lower than that of some engineered biochars, AlBC’s consistent performance across diverse conditions, combined with its low-cost waste-derived origin, highlights its potential as a sustainable and effective adsorbent for the removal of emerging contaminants in water treatment applications.

## Figures and Tables

**Table 1:** Kinetic parameters of CAF, CBZ, and EE2 adsorption on AlBCSS.

Compound		Pseudo-First Order			Pseudo-Second Order			Elovich		
	q_e,exp_	q_e,cal_	K_1_	R^2^	q_e,cal_	K_2_	R^2^	α	β	R^2^
	mg g^−1^	mg g^−1^	min^−1^		mg g^−1^	g mg^−1^ min^−1^		mg g^−1^ min^−1^	g mg^−1^	
CAF	4.58	4.58	0.021	0.694	4.61	0.0306	0.998	51.936	2.278	0.996
CBZ	6.12	5.81	0.073	0.977	6.14	0.018	0.996	18.077	1.469	0.998
EE2	9.51	9.15	0.094	0.980	9.53	0.017	0.994	253.87	1.195	0.9987

**Table 2:** Parameters from the isotherms study of CAF, CBZ, and EE2 adsorption on AlBCSS.

Compound	Langmuir			Freundlich		
	q_max_	K_L_	R^2^	K_F_	1/n_F_	R^2^
K	mg g^−1^	L mg^−1^		mg g^−1^ (mg^−1^)^n^		
CAF	17.05	0.071	0.995	1.771	0.591	0.988
CBZ	16.59	0.161	0.973	3.485	0.455	0.951
EE2	23.61	1.196	0.685	13.251	0.262	0.930

**Table 3:** Thermodynamic parameters for the adsorption of CAF, CBZ, and EE2 on AlBCSS at 10, 25, and 40 °C.

Temperature	Caffeine			Carbamazepine			17 α-Ethinylestradiol		
	ΔG^0^	ΔH^0^	ΔS^0^	ΔG^0^	ΔH^0^	ΔS^0^	ΔG^0^	ΔH^0^	ΔS^0^
K	KJ mol^−1^	KJ mol^−1^	J (mol K)^−1^	KJ mol^−1^	KJ mol^−1^	J (mol K)^−1^	KJ mol^−1^	KJ mol^−1^	J (mol K)^−1^
283	−33.07	−15.99	60.32	−34.24	2.30	131.48	−46.78	3.09	176.25
298	−33.97			−36.21			−49.43		
313	−34.88			−38.19			−52.07		

**Table 4:** Comparison of the performance of AlBC with that of other carbon-based adsorbents for the removal of CAF, CBZ, and EE2.

Contaminant	Adsorbent Medium	Feedstock	Specific Surface Area	Conditions	Maximum Adsorption Capacity	Ref.
**CAF**	Biochar	Gliricidia sepium	216.40 m^2^ g^−1^	C_0_: 10–500 mg L^−1^	16.26 mg g^−1^	[80]
				C_biochar_: 1 g L^−1^		
				t: 24 h		
	Steam-activated biochar	Tea waste	576.09 m^2^ g^−1^	C_0_: 10–300 mg L^−1^	15.4 mg g^−1^	[81]
				C_biochar_: 1 g L^−1^		
				t: 24 h		
	Ball-milled biochar	Spent coffee ground (SCG)	10.114 m^2^ g^−1^	C_0_: 5–200 mg L^−1^	82.65 mg g^−1^	[82]
				C_biochar_: 1 g L^−1^		
				t: 24 h		
	AlBC	Sewage sludge	86.41 m^2^ g^−1^	C_0_: 7–28 mg L^−1^	17.05 mg g^−1^	This study
				C_biochar_: 1 g L^−1^		
				t: 6 h		
**CBZ**	Powdered activated Carbon	Vegetable	1328 m^2^ g^−1^	C_0_: 10–40 mg L^−1^	220 mg g^−1^	[83]
				C_biochar_: 0.1 g L^−1^		
				t: 72 h		
	K_2_CO_3_ phosphoric acid-modified hydrochar	Pine sawdust	1265.08 m^2^ g^−1^	C_0_: 30–70 mg L^−1^	376.1 mg g^−1^	[78]
				C_biochar_: 0.1 g L^−1^		
				t: 24 h		
	Ppy-GO-Biochar Nanocomposite	Palm seeds	8.5983 m^2^ g^−1^	C_0_: -	45 mg g^−1^	[84]
				C_biochar_: 1.4 g L^−1^		
				t: 5.5 h		
	AlBC	Sewage sludge	86.41 m^2^ g^−1^	C_0_: 7–28 mg L^−1^	16.59 mg g^−1^	This study
				C_biochar_: 1 g L^−1^		
				t: 6 h		
**EE2**	Biochar	Pumpkin	8.69 m^2^ g^−1^	C_0_: 2–20 mg L^−1^	66.26 mg g^−1^	[85]
				C_biochar_: 0.8 g L^−1^		
				t: 2 h		
	Modified activated carbon cloths	Viscose rayon cloth	820 m^2^ g^−1^	C_0_: 2–12 mg L^−1^	11.11 mg g^−1^	[86]
				C_biochar_: 0.8 g L^−1^		
				t: 24 h		
	Biochar	Corn straw	298 m^2^ g^−1^	C_0_: 0.2–4 mg L^−1^	1696 µg g^−1^	[87]
				C_biochar_: 1 g L^−1^		
				t: 48 h		
	AlBC	Sewage sludge	86.41 m^2^ g^−1^	C_0_: 7–28 mg L^−1^	23.61 mg g^−1^	This study
				C_biochar_: 1 g L^−1^		
				t: 6 h		

**Table 5:** Chemical structures and physicochemical properties of the studied contaminants.

Compound	Structure	Molecular Formula	Molecular Mass	pKa		LogKow	Solubility
							in H_2_O
			g mol^−1^	1	2		g L^−1^
Caffeine (CAF)	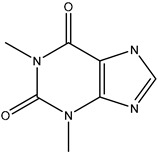	C_8_H_10_N_4_O_2_	194.2	0.7	14	−0.091	20
Carbamazepine (CBZ)	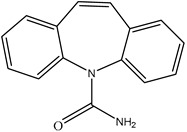	C_15_H_12_N_2_O	236.3	1	13.9	2.45	0.152
17 α- ethinylestradiol (EE2)	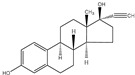	C_20_H_24_O_2_	296.4	10.4	-	3.67	0.011

## Data Availability

Data available upon request.

## Supplementary Materials

The following supporting information can be downloaded at https://www.mdpi.com/article/10.3390/molecules30173514/s1, Figure S1: Brunauer–Emmett–Teller (BET) measurements with N_2_ adsorption and desorption isotherms of (a) BC and (b) AlBC; Figure S2: Fluorescence (a) and UV-Vis (b) spectra of the river sample; Figure S3: ATR-FTIR of AlBC before and after the adsorption of CAF, CBZ, and EE2; Table S1: EDS characterization of BC; Table S2: Characterization of BC and AlBC; Table S3: Comparison of the different applied kinetic models based on the adjusted R^2^, Residual Sum of Squares, and Reduced Chi-Square values; Table S4: Comparison of the different applied isotherm models based on the adjusted R^2^, Residual Sum of Squares, and Reduced Chi-Square values; Table S5: Physicochemical parameters of the natural water sample; Table S6: Validation parameters of the applied HPLC-DAD method for the analysis of the studied contaminants.

## Abbreviations

The following abbreviations are used in this manuscript:

WWTPs	Wastewater Treatment Plants
BC	Sewage Sludge-Derived Biochar
AlBC	Alkaline-Treated Sewage Sludge-Derived Biochar
CAF	Caffeine
CBZ	Carbamazepine
EE2	17α-ethinyl estradiol
PhACs	Pharmaceutical compounds
EDCs	Endocrine-disrupting chemicals
BET	Brunauer–Emmett–Teller
SEM	Scanning electron microscopy
ATR-FTIR	Attenuated Total Reflection Fourier-Transform Infrared Spectroscopy
LDE	Laser Doppler Electrophoresis
TOC	Total Organic Carbon
IC	Inorganic Carbon
UV-Vis	Ultraviolet–Visible
NOM	Natural Organic Matter
HPLC-DAD	High-Performance Liquid Chromatography with a Diode Array Detector
ACN	Acetonitrile
PFO	Pseudo-First Order
PSO	Pseudo-Second Order
DI water	Deionized water

## References

[1] Eurostat 2022, Sewage Sludge Production and Disposal. https://ec.europa.eu/eurostat/databrowser/view/env_ww_spd/default/table?lang=en.

[2] Bondarczuk K., Markowicz A., Piotrowska-Seget Z. (2016). The Urgent Need for Risk Assessment on the Antibiotic Resistance Spread via Sewage Sludge Land Application. Environ. Int..

[3] Kacprzak M., Neczaj E., Fijałkowski K., Grobelak A., Grosser A., Worwag M., Rorat A., Brattebo H., Almås Å., Singh B.R. (2017). Sewage Sludge Disposal Strategies for Sustainable Development. Environ. Res..

[4] Regkouzas P., Diamadopoulos E. (2019). Adsorption of Selected Organic Micro-Pollutants on Sewage Sludge Biochar. Chemosphere.

[5] Chaudhary H., Dinakaran J., Rao K.S. (2024). Comparative Analysis of Biochar Production Methods and Their Impacts on Biochar Physico-Chemical Properties and Adsorption of Heavy Metals. J. Environ. Chem. Eng..

[6] Gopinath A., Divyapriya G., Srivastava V., Laiju A.R., Nidheesh P.V., Kumar M.S. (2021). Conversion of Sewage Sludge into Biochar: A Potential Resource in Water and Wastewater Treatment. Environ. Res..

[7] Zhao L., Sun Z.-F., Pan X.-W., Tan J.-Y., Yang S.-S., Wu J.-T., Chen C., Yuan Y., Ren N.-Q. (2023). Sewage Sludge Derived Biochar for Environmental Improvement: Advances, Challenges, and Solutions. Water Res. X.

[8] Pipíška M., Krajčíková E.K., Hvostik M., Frišták V., Ďuriška L., Černičková I., Kaňuchová M., Conte P., Soja G. (2022). Biochar from Wood Chips and Corn Cobs for Adsorption of Thioflavin T and Erythrosine B. Materials.

[9] Zafeer M.K., Menezes R.A., Venkatachalam H., Bhat K.S. (2024). Sugarcane Bagasse-Based Biochar and Its Potential Applications: A Review. Emergent Mater..

[10] Torres Castillo N.E., Ochoa Sierra J.S., Oyervides-Muñoz M.A., Sosa-Hernández J.E., Iqbal H.M.N., Parra-Saldívar R., Melchor-Martínez E.M. (2021). Exploring the Potential of Coffee Husk as Caffeine Bio-Adsorbent—A Mini-Review. Case Stud. Chem. Environ. Eng..

[11] Agrafioti E., Kalderis D., Diamadopoulos E. (2014). Arsenic and Chromium Removal from Water Using Biochars Derived from Rice Husk, Organic Solid Wastes and Sewage Sludge. J. Environ. Manag..

[12] Krounbi L., Enders A., Gaunt J., Ball M., Lehmann J. (2021). Plant Uptake of Nitrogen Adsorbed to Biochars Made from Dairy Manure. Sci. Rep..

[13] Zheng W., Guo M., Chow T., Bennett D.N., Rajagopalan N. (2010). Sorption Properties of Greenwaste Biochar for Two Triazine Pesticides. J. Hazard. Mater..

[14] Hu J., Zhao L., Luo J., Gong H., Zhu N. (2022). A Sustainable Reuse Strategy of Converting Waste Activated Sludge into Biochar for Contaminants Removal from Water: Modifications, Applications and Perspectives. J. Hazard. Mater..

[15] Jung C., Park J., Lim K.H., Park S., Heo J., Her N., Oh J., Yun S., Yoon Y. (2013). Adsorption of Selected Endocrine Disrupting Compounds and Pharmaceuticals on Activated Biochars. J. Hazard. Mater..

[16] Liu Z., Singer S., Tong Y., Kimbell L., Anderson E., Hughes M., Zitomer D., McNamara P. (2018). Characteristics and Applications of Biochars Derived from Wastewater Solids. Renew. Sustain. Energy Rev..

[17] Wang J., Wang S. (2019). Preparation, Modification and Environmental Application of Biochar: A Review. J. Clean. Prod..

[18] Cui J., Wei L., Ning C., Zhang F., Cui J., Xiangli P. (2024). Highly-Efficient Persulfate Activation by Nitrogen-Doped Biochar Derived from Dewatered Sewage Sludge for 2,4-Dichlorophenol Removal: Process and Mechanism. J. Environ. Chem. Eng..

[19] Chen L., Yang H., Hong R., Xie X., Zuo R., Zhang X., Chen S., Xu D., Zhang Q. (2024). Tetracycline Adsorption on Sludge-Bamboo Biochar Prepared by Gradient Modification and Co-Pyrolysis: Performance Evaluation and Mechanism Insight. J. Environ. Chem. Eng..

[20] Wang J., Liao Z., Ifthikar J., Shi L., Chen Z., Chen Z. (2017). One-Step Preparation and Application of Magnetic Sludge-Derived Biochar on Acid Orange 7 Removal via Both Adsorption and Persulfate Based Oxidation. RSC Adv..

[21] Pandey D., Daverey A., Arunachalam K. (2020). Biochar: Production, Properties and Emerging Role as a Support for Enzyme Immobilization. J. Clean. Prod..

[22] Shahib I.I., Ifthikar J., Oyekunle D.T., Elkhlifi Z., Jawad A., Wang J., Lei W., Chen Z. (2022). Influences of Chemical Treatment on Sludge Derived Biochar; Physicochemical Properties and Potential Sorption Mechanisms of Lead (II) and Methylene Blue. J. Environ. Chem. Eng..

[23] Tran N.H., Reinhard M., Gin K.Y.-H. (2018). Occurrence and Fate of Emerging Contaminants in Municipal Wastewater Treatment Plants from Different Geographical Regions-a Review. Water Res..

[24] Quadra G.R., Paranaíba J.R., Vilas-Boas J., Roland F., Amado A.M., Barros N., Dias R.J.P., Cardoso S.J. (2020). A Global Trend of Caffeine Consumption over Time and Related-Environmental Impacts. Environ. Pollut..

[25] Wikoff D., Welsh B.T., Henderson R., Brorby G.P., Britt J., Myers E., Goldberger J., Lieberman H.R., O’Brien C., Peck J. (2017). Systematic Review of the Potential Adverse Effects of Caffeine Consumption in Healthy Adults, Pregnant Women, Adolescents, and Children. Food Chem. Toxicol..

[26] Santos-Silva T.G., Montagner C.C., Martinez C.B.R. (2018). Evaluation of Caffeine Effects on Biochemical and Genotoxic Biomarkers in the Neotropical Freshwater Teleost Prochilodus lineatus. Environ. Toxicol. Pharmacol..

[27] Bogusz A., Tomczyk B., Trzcińska M., Mirosław B., Gworek B. (2024). Effect of Zeolites on the Reduction of the Ecotoxicity of Carbamazepine in the Environment. Ecotoxicol. Environ. Saf..

[28] Almeida Â., Soares A.M.V.M., Esteves V.I., Freitas R. (2021). Occurrence of the Antiepileptic Carbamazepine in Water and Bivalves from Marine Environments: A Review. Environ. Toxicol. Pharmacol..

[29] Zhou X., Zhou J., Liu Y., He Y., Ren J., Guo J. (2019). Adsorption of Endocrine Disrupting Ethylparaben from Aqueous Solution by Chemically Activated Biochar Developed from Oil Palm Fibre. Sep. Sci. Technol..

[30] Ewis D., Ba-Abbad M.M., Benamor A., El-Naas M.H. (2022). Adsorption of Organic Water Pollutants by Clays and Clay Minerals Composites: A Comprehensive Review. Appl. Clay Sci..

[31] Ibrahim A.O., Adegoke K.A., Adegoke R.O., AbdulWahab Y.A., Oyelami V.B., Adesina M.O. (2021). Adsorptive Removal of Different Pollutants Using Metal-Organic Framework Adsorbents. J. Mol. Liq..

[32] Thompson K.A., Shimabuku K.K., Kearns J.P., Knappe D.R.U., Summers R.S., Cook S.M. (2016). Environmental Comparison of Biochar and Activated Carbon for Tertiary Wastewater Treatment. Environ. Sci. Technol..

[33] Li B., Yang L., Wang C.-Q., Zhang Q.-P., Liu Q.-C., Li Y.-D., Xiao R. (2017). Adsorption of Cd(II) from Aqueous Solutions by Rape Straw Biochar Derived from Different Modification Processes. Chemosphere.

[34] Yin Q., Liu M., Ren H. (2019). Biochar Produced from the Co-Pyrolysis of Sewage Sludge and Walnut Shell for Ammonium and Phosphate Adsorption from Water. J. Environ. Manag..

[35] Yuan H., Lu T., Huang H., Zhao D., Kobayashi N., Chen Y. (2015). Influence of Pyrolysis Temperature on Physical and Chemical Properties of Biochar Made from Sewage Sludge. J. Anal. Appl. Pyrolysis.

[36] Hossain M.K., Strezov V., Chan K.Y., Ziolkowski A., Nelson P.F. (2011). Influence of Pyrolysis Temperature on Production and Nutrient Properties of Wastewater Sludge Biochar. J. Environ. Manag..

[37] Jin J., Li Y., Zhang J., Wu S., Cao Y., Liang P., Zhang J., Wong M.H., Wang M., Shan S. (2016). Influence of Pyrolysis Temperature on Properties and Environmental Safety of Heavy Metals in Biochars Derived from Municipal Sewage Sludge. J. Hazard. Mater..

[38] Raj A., Yadav A., Rawat A.P., Singh A.K., Kumar S., Pandey A.K., Sirohi R., Pandey A. (2021). Kinetic and Thermodynamic Investigations of Sewage Sludge Biochar in Removal of Remazol Brilliant Blue R Dye from Aqueous Solution and Evaluation of Residual Dyes Cytotoxicity. Environ. Technol. Innov..

[39] Zhang Y., Maierdan Y., Guo T., Chen B., Fang S., Zhao L. (2022). Biochar as Carbon Sequestration Material Combines with Sewage Sludge Incineration Ash to Prepare Lightweight Concrete. Constr. Build. Mater..

[40] Kalderis D., Kayan B., Akay S., Kulaksız E., Gözmen B. (2017). Adsorption of 2,4-Dichlorophenol on Paper Sludge/Wheat Husk Biochar: Process Optimization and Comparison with Biochars Prepared from Wood Chips, Sewage Sludge and Hog Fuel/Demolition Waste. J. Environ. Chem. Eng..

[41] Feng Z., Zhu L. (2018). Sorption of Phenanthrene to Biochar Modified by Base. Front. Environ. Sci. Eng..

[42] Tang G., Mo H., Gao L., Chen Y., Zhou X. (2024). Adsorption of Crystal Violet from Wastewater Using Alkaline-Modified Pomelo Peel-Derived Biochar. J. Water Process Eng..

[43] Zhao H., Wang Z., Liang Y., Wu T., Chen Y., Yan J., Zhu Y., Ding D. (2023). Adsorptive Decontamination of Antibiotics from Livestock Wastewater by Using Alkaline-Modified Biochar. Environ. Res..

[44] Shin J., Kwak J., Lee Y.-G., Kim S., Son C., Cho K.H., Lee S.-H., Park Y., Ren X., Chon K. (2021). Changes in Adsorption Mechanisms of Radioactive Barium, Cobalt, and Strontium Ions Using Spent Coffee Waste Biochars via Alkaline Chemical Activation: Enrichment Effects of O-Containing Functional Groups. Environ. Res..

[45] Yan Q., Nosratabad N.A., Du X., Ketelboeter T., Wan C., Cai Z. (2025). Highly Effective Lead Removal by Novel Alkaline Biochar Prepared by Pyrolysis of Woody Biomass Impregnated with Low-Level NaOH. J. Hazard. Mater. Adv..

[46] Liu C., Wang W., Wu R., Liu Y., Lin X., Kan H., Zheng Y. (2020). Preparation of Acid- and Alkali-Modified Biochar for Removal of Methylene Blue Pigment. ACS Omega.

[47] Yang H., Li X., Wang Y., Wang J., Yang L., Ma Z., Luo J., Cui X., Yan B., Chen G. (2023). Effective Removal of Ammonium from Aqueous Solution by Ball-Milled Biochar Modified with NaOH. Processes.

[48] Anastopoulos I., Katsouromalli A., Pashalidis I. (2020). Oxidized Biochar Obtained from Pine Needles as a Novel Adsorbent to Remove Caffeine from Aqueous Solutions. J. Mol. Liq..

[49] Dos Santos D.F., Moreira W.M., De Araújo T.P., Bernardo M.M.S., de Figueiredo Ligeiro da Fonseca I.M., Ostroski I.C., De Barros M.A.S.D. (2023). Competitive Adsorption of Acetaminophen and Caffeine onto Activated *Tingui* Biochar: Characterization, Modeling, and Mechanisms. Environ. Sci. Pollut. Res..

[50] Beltrame K.K., Cazetta A.L., De Souza P.S.C., Spessato L., Silva T.L., Almeida V.C. (2018). Adsorption of Caffeine on Mesoporous Activated Carbon Fibers Prepared from Pineapple Plant Leaves. Ecotoxicol. Environ. Saf..

[51] Hama Aziz K.H., Mustafa F.S., Hassan M.A., Omer K.M., Hama S. (2024). Biochar as Green Adsorbents for Pharmaceutical Pollution in Aquatic Environments: A Review. Desalination.

[52] Zhang W., Sheng X., Yan J., Wang J., Sun J., Zuo Q., Zhu X., Wang M., Gong L. (2023). The Crucial Role of Different NaOH Activation Pathways on the Algae-Derived Biochar toward Carbamazepine Adsorption. Results Eng..

[53] Shin J., Lee Y.-G., Lee S.-H., Kim S., Ochir D., Park Y., Kim J., Chon K. (2020). Single and Competitive Adsorptions of Micropollutants Using Pristine and Alkali-Modified Biochars from Spent Coffee Grounds. J. Hazard. Mater..

[54] Cuong Nguyen X., Thanh Huyen Nguyen T., Hong Chuong Nguyen T., Van Le Q., Yen Binh Vo T., Cuc Phuong Tran T., Duong La D., Kumar G., Khanh Nguyen V., Chang S.W. (2021). Sustainable Carbonaceous Biochar Adsorbents Derived from Agro-Wastes and Invasive Plants for Cation Dye Adsorption from Water. Chemosphere.

[55] Zanella H.G., Spessato L., Lopes G.K.P., Yokoyama J.T.C., Silva M.C., Souza P.S.C., Ronix A., Cazetta A.L., Almeida V.C. (2021). Caffeine Adsorption on Activated Biochar Derived from Macrophytes (*Eichornia crassipes*). J. Mol. Liq..

[56] Ahmed M.B., Zhou J.L., Ngo H.H., Johir M.A.H., Sornalingam K. (2018). Sorptive Removal of Phenolic Endocrine Disruptors by Functionalized Biochar: Competitive Interaction Mechanism, Removal Efficacy and Application in Wastewater. Chem. Eng. J..

[57] Oginni O., Singh K. (2021). Effect of Carbonization Temperature on Fuel and Caffeine Adsorption Characteristics of White Pine and Norway Spruce Needle Derived Biochars. Ind. Crops Prod..

[58] Całus-Makowska K., Grosser A., Grobelak A., Białek H., Siedlecka E. (2024). Kinetic Study of the Simultaneous Removal of Ibuprofen, Carbamazepine, Sulfamethoxazole, and Diclofenac from Water Using Biochar and Activated Carbon Adsorption, and TiO_2_ Photocatalysis. Desalination Water Treat..

[59] Décima M.A., Marzeddu S., Barchiesi M., Di Marcantonio C., Chiavola A., Boni M.R. (2021). A Review on the Removal of Carbamazepine from Aqueous Solution by Using Activated Carbon and Biochar. Sustainability.

[60] Quintero-Jaramillo J.A., Carrero-Mantilla J.I., Sanabria-González N.R. (2021). A Review of Caffeine Adsorption Studies onto Various Types of Adsorbents. Sci. World J..

[61] Mao J., Zhang K., Chen B. (2019). Linking Hydrophobicity of Biochar to the Water Repellency and Water Holding Capacity of Biochar-Amended Soil. Environ. Pollut..

[62] Lai C., He H., Xie W., Fan S., Huang H., Wang Y., Huang B., Pan X. (2022). Adsorption and Photochemical Capacity on 17α-Ethinylestradiol by Char Produced in the Thermo Treatment Process of Plastic Waste. J. Hazard. Mater..

[63] Ahmed M.B., Zhou J.L., Ngo H.H., Johir M.A.H., Sun L., Asadullah M., Belhaj D. (2018). Sorption of Hydrophobic Organic Contaminants on Functionalized Biochar: Protagonist Role of π-π Electron-Donor-Acceptor Interactions and Hydrogen Bonds. J. Hazard. Mater..

[64] Vieira R.A.L., Pickler T.B., Segato T.C.M., Jozala A.F., Grotto D. (2022). Biochar from Fungiculture Waste for Adsorption of Endocrine Disruptors in Water. Sci. Rep..

[65] Anastopoulos I., Kyzas G.Z. (2016). Are the Thermodynamic Parameters Correctly Estimated in Liquid-Phase Adsorption Phenomena?. J. Mol. Liq..

[66] Melo L.L.A., Ide A.H., Duarte J.L.S., Zanta C.L.P.S., Oliveira L.M.T.M., Pimentel W.R.O., Meili L. (2020). Caffeine Removal Using Elaeis Guineensis Activated Carbon: Adsorption and RSM Studies. Environ. Sci. Pollut. Res..

[67] Zayyat R.M., Yahfoufi R., Al-Hindi M., Kordahi M.A., Ayoub G.M., Ahmad M.N. (2024). Elucidating the Dynamics of Carbamazepine Uptake Using Date Pit-Derived Activated Carbon: A Comprehensive Kinetic and Thermodynamic Analysis. Heliyon.

[68] Kumar D., Pandey L.K., Gaur J.P. (2016). Metal Sorption by Algal Biomass: From Batch to Continuous System. Algal Res..

[69] Fan S., Wang Y., Wang Z., Tang J., Tang J., Li X. (2017). Removal of Methylene Blue from Aqueous Solution by Sewage Sludge-Derived Biochar: Adsorption Kinetics, Equilibrium, Thermodynamics and Mechanism. J. Environ. Chem. Eng..

[70] Zeghioud H., Fryda L., Mahieu A., Visser R., Kane A. (2022). Potential of Flax Shives and Beech Wood-Derived Biochar in Methylene Blue and Carbamazepine Removal from Aqueous Solutions. Materials.

[71] Barquilha C.E.R., Braga M.C.B. (2021). Adsorption of Organic and Inorganic Pollutants onto Biochars: Challenges, Operating Conditions, and Mechanisms. Bioresour. Technol. Rep..

[72] Ngeno E.C., Orata F., Baraza L.D., Shikuku V.O., Kimosop S.J. (2016). Adsorption of Caffeine and Ciprofloxacin onto Pyrolitically Derived Water Hyacinth Biochar: Isothermal, Kinetic and Thermodynamic Studies. J. Chem. Chem. Eng..

[73] Anastopoulos I., Pashalidis I. (2019). Τhe Application of Oxidized Carbon Derived from *Luffa cylindrica* for Caffeine Removal. Equilibrium, Thermodynamic, Kinetic and Mechanistic Analysis. J. Mol. Liq..

[74] Prokic D., Vukčević M., Mitrović A., Maletić M., Kalijadis A., Častvan I.J., Đurkić T. (2022). Adsorption of Estrone, 17β-Estradiol, and 17α-Ethinylestradiol from Water onto Modified Multi-Walled Carbon Nanotubes, Carbon Cryogel, and Carbonized Hydrothermal Carbon. Environ. Sci. Pollut. Res..

[75] Alsawy T., Rashad E., El-Qelish M., Mohammed R.H. (2022). A Comprehensive Review on the Chemical Regeneration of Biochar Adsorbent for Sustainable Wastewater Treatment. npj Clean Water.

[76] Behera A.K., Shadangi K.P., Sarangi P.K. (2024). Efficient Removal of Rhodamine B Dye Using Biochar as an Adsorbent: Study the Performance, Kinetics, Thermodynamics, Adsorption Isotherms and Its Reusability. Chemosphere.

[77] Francoeur M., Ferino-Pérez A., Yacou C., Jean-Marius C., Emmanuel E., Chérémond Y., Jauregui-Haza U., Gaspard S. (2021). Activated Carbon Synthetized from *Sargassum* (*sp*) for Adsorption of Caffeine: Understanding the Adsorption Mechanism Using Molecular Modeling. J. Environ. Chem. Eng..

[78] Zhong H., Zhu G., Wang Z., Liu X., Zhang H., Qiu Y., Yin D., Zhu Z. (2025). Efficient Adsorption Removal of Carbamazepine from Water by Dual-Activator Modified Hydrochar. Sep. Purif. Technol..

[79] Regkouzas P., Sygellou L., Diamadopoulos E. (2025). Emerging Micro-Contaminant Adsorption from Water and Wastewater Using CNT-Doped Biochar Nanocomposites from Rice Husks and Sewage Sludge. Biomass Convers. Biorefinery.

[80] Keerthanan S., Rajapaksha S.M., Trakal L., Vithanage M. (2020). Caffeine Removal by *Gliricidia sepium* Biochar: Influence of Pyrolysis Temperature and Physicochemical Properties. Environ. Res..

[81] Keerthanan S., Bhatnagar A., Mahatantila K., Jayasinghe C., Ok Y.S., Vithanage M. (2020). Engineered Tea-Waste Biochar for the Removal of Caffeine, a Model Compound in Pharmaceuticals and Personal Care Products (PPCPs), from Aqueous Media. Environ. Technol. Innov..

[82] Yang Y., Wan Y., Chen J., Chen H., Li Y., Muñoz-Carpena R., Zheng Y., Huang J., Zhang Y., Gao B. (2025). Ball-Milled Spent Coffee Ground Biochar Effectively Removes Caffeine from Water. Water.

[83] Delgado N., Capparelli A., Navarro A., Marino D. (2019). Pharmaceutical Emerging Pollutants Removal from Water Using Powdered Activated Carbon: Study of Kinetics and Adsorption Equilibrium. J. Environ. Manag..

[84] Agilandeswari P., Venkateshbabu S., Sarojini G., Rajasimman M. (2024). Sustainable Development and Analysis of a Novel Bio-Derived (Biochar) Nanocomposite for the Remediation of Carbamazepine from Aqueous Solution. Chemosphere.

[85] Barkahoum B., Saadia G., Asma N. (2025). Removal of Active Pharmaceutical Compounds in Primalan and Diane Using Pumpkin Biochar: Synthesis, Characterization, and Adsorption Study. RSC Adv..

[86] Prokić D., Vukčević M., Kalijadis A., Maletić M., Babić B., Đurkić T. (2020). Removal of Estrone, 17β-Estradiol, and 17α-Ethinylestradiol from Water by Adsorption onto Chemically Modified Activated Carbon Cloths. Fibers Polym..

[87] Guo W., Lu S., Shi J., Zhao X. (2019). Effect of Corn Straw Biochar Application to Sediments on the Adsorption of 17α-Ethinyl Estradiol and Perfluorooctane Sulfonate at Sediment-Water Interface. Ecotoxicol. Environ. Saf..

[88] Wu F.-C., Tseng R.-L., Juang R.-S. (2009). Characteristics of Elovich Equation Used for the Analysis of Adsorption Kinetics in Dye-Chitosan Systems. Chem. Eng. J..

[89] Ho Y.S., McKay G. (1999). Pseudo-Second Order Model for Sorption Processes. Process Biochem..

[90] Langmuir I. (1916). The Constitution and Fundamental Properties of Solids and Liquids. Part I. Solids. J. Am. Chem. Soc..

[91] Freundlich H. (1907). Über Die Adsorption in Lösungen. Z. Phys. Chem..

[92] Zhou X., Zhou X. (2014). The Unit Problem in the Thermodynamic Calculation of Adsorption Using the Langmuir Equation. Chem. Eng. Commun..

