# Supplementary material for: Activated Biochar from Sewage Sludge: A Sustainable Solution for Effective Removal of Emerging Water Contaminants
Source: Molecules. 2025 Aug 28;30(17):3514. doi: 10.3390/molecules30173514 (PMC12430717; doi:10.3390/molecules30173514)
Supplement: Supplementary file 1 [file molecules-30-03514-s001.zip › molecules-3754885-supplementary.pdf]

molecules

Article

Activated Biochar from Sewage Sludge: A Sustainable Solution for Effective Removal of Emerging Water Contaminants

Marina Anastasiou [1,2], Vasilios Sakkas [2] and Mohamad Sleiman [1,*]

[1] Institute of Chemistry of Clermont Ferrand, Universite Clermont Auvergne, Clermont Auvergne INP, CNRS, ICCF, F-63000 Clermont-Ferrand, France; marina.anastasiou@sigma-clermont.fr
[2] Department of Chemistry, University of Ioannina, 45110 Ioannina, Greece; vsakkas@uoi.gr
* Correspondence: mohamad.sleiman@sigma-clermont.fr

Academic Editor: M. Gilles Mailhot

Received: 27 June 2025
Revised: 28 July 2025
Accepted: 28 July 2025
Published: 28 August 2025

Citation: Anastasiou, M.; Sakkas, V.; Sleiman, M. Activated Biochar from Sewage Sludge: A Sustainable Solution for Effective Removal of EmergingWater Contaminants. Molecules **2025**, *30*, 3514. https://doi.org/10.3390/molecules30173514

Copyright: © 2025 by the authors. Licensee MDPI, Basel, Switzerland. This article is an open access article distributed under the terms and conditions of the Creative Commons Attribution (CC BY) license (https://creativecommons.org/licenses/by/4.0/).

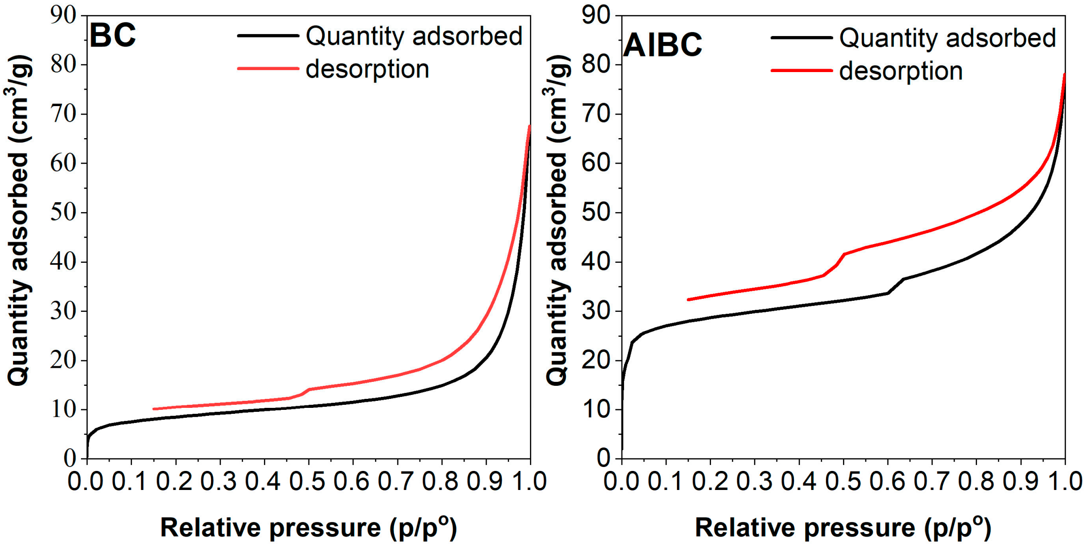

Figure S1. Brunner-Emmet-Teller (BET) measurements with N_2 adsorption and desorption isotherms of (a) BC and (b) AlBC.

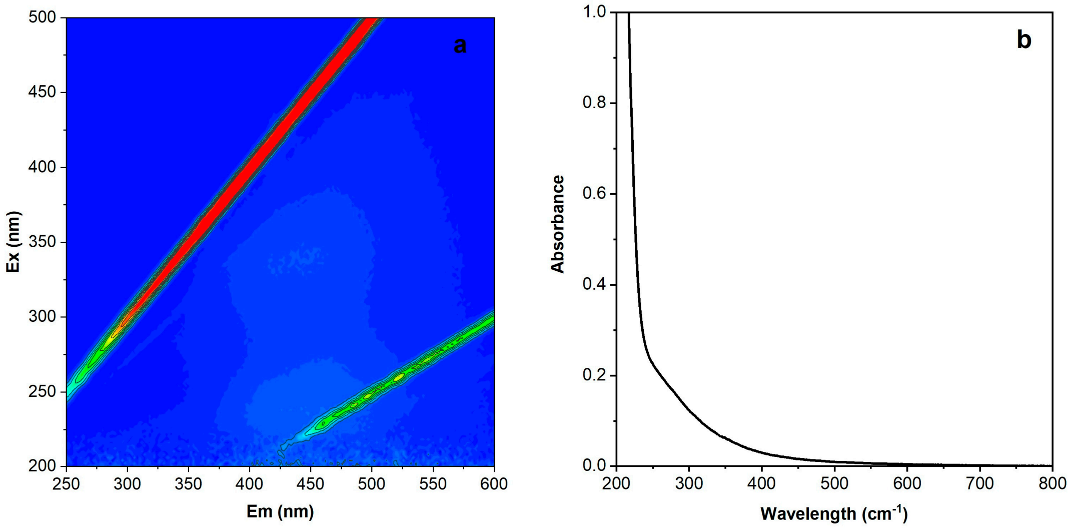

Figure S2. Fluorescence (a) and UV-Vis (b) spectra of the river sample.

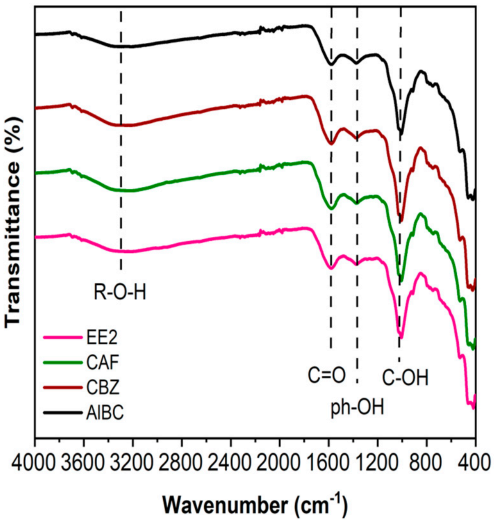

Figure S3. ATR-FTIR of AlBC before and after the adsorption of CAF, CBZ and EE2

Table S1. EDS characterization of BC.

**Element**	**A**	**Ray**	**Net**	**Mass [%]**	**Mass norm. [%]**	**Atom. [%]**	**Error abs. [%] (1 sigma)**	**Error abs. [%] (2 sigma)**	**Error abs. [%] (3 sigma)**
**C**	6	K	37734	55.17	55.17	65.09	3.04	6.08	9.12
**O**	8	K	29481	34.87	34.87	30.88	1.93	3.86	5.78
**Al**	13	K	25526	1.57	1.57	0.83	0.07	0.13	0.20
**Si**	14	K	63255	2.69	2.69	1.36	0.10	0.20	0.30
**P**	15	K	22993	1.13	1.13	0.52	0.04	0.07	0.11
**S**	16	K	5312	0.25	0.25	0.11	0.01	0.02	0.03
**Ca**	20	K	21695	1.29	1.29	0.46	0.03	0.07	0.10
**Fe**	26	K	27072	3.03	3.03	0.77	0.07	0.14	0.21
			**Total**	100.00	100.00				

Table S2. Characterization of BC and AlBC.

**Material**	**Particle Size (DX 50)** μm	**pH**	**Zeta Potential** mV	**Surface Area** $m^2\ g^{-1}$	**Pore Volume** $cm^3\ g^{-1}$
BC	8.19	7.10	-26.18	28.85	0.0042
AlBC	24.1	7.69	-32.69	86.41	0.0295

Table S3. Comparison of the different applied kinetic models based on the adjusted R^2, Residual Sum of Squares and Reduced Chi-square values.

Contaminant	Statistical Parameters	PFO	PSO	Elovich
CAF	Adjusted R^2	0.694	0.998	0.996
	Residual Sum of Squares	4.47	0.024	0.050
	Reduced Chi-square	0.74	0.0039	0.0083
CBZ	Adjusted R^2	0.977	0.996	0.997
	Residual Sum of Squares	0.59	0.099	0.05835
	Reduced Chi-square	0.098	0.016	0.0097
EE2	Adjusted R^2	0.979	0.994	0.998
	Residual Sum of Squares	1.28	0.376	0.081
	Reduced Chi-square	0.21	0.063	0.0135

Table S4. Comparison of the different applied Isotherms models based on the adjusted R^2, Residual Sum of Squares and Reduced Chi-square values.

Contaminant	Statistical Parameters	Langmuir	Freundlich
**CAF**	Adjusted R^2	0,994	0,988
	Residual Sum of Squares	0,127	0,291
	Reduced Chi-square	0,025	0,058
**CBZ**	Adjusted R^2	0,977	0,951
	Residual Sum of Squares	0,947	1,736
	Reduced Chi-square	0,189	0,347
**EE2**	Adjusted R^2	0,685	0,929
	Residual Sum of Squares	45,69	10,21
	Reduced Chi-square	9,14	2,04

Table S5. Physicochemical parameters of the natural water sample.

Sample	pH	Turbidity	TOC	IC	OC
		NTU	mg L^{-1}	mg L^{-1}	mg L^{-1}
River	7.86	5.62	30.95	23.83	7.12

Table S6. Validation parameters of the applied HPLC-DAD method for the analysis of the studied contaminants.

Contaminant	LDR[1] mg L^{-1}	Regression Equation	R^2	LOD mg L^{-1}	LOQ mg L^{-1}
CAF	0.5-50	y=16198.54*x + 1075.52	0.999	0.35	1.08
CBZ	0.5-50	y=16105.49*x -1790.89	0.998	2.32	7.03
EE2	0.5-28	y=1808.65*x + 954.57	0.994	2.50	7.56

[1] LDR- Linear dynamic range
